# A Tetraperylene Diimides Based 3D Nonfullerene Acceptor for Efficient Organic Photovoltaics

**DOI:** 10.1002/advs.201500014

**Published:** 2015-02-27

**Authors:** Shi‐Yong Liu, Chen‐Hao Wu, Chang‐Zhi Li, Sheng‐Qiang Liu, Kung‐Hwa Wei, Hong‐Zheng Chen, Alex K.‐Y. Jen

**Affiliations:** ^1^ Department of Materials Science and EngineeringUniversity of WashingtonBox 352120SeattleWA98195USA; ^2^ Department of Polymer Science and EngineeringZhejiang UniversityHangzhou310027P.R. China; ^3^ Department of Pharmacy and ChemistryTaizhou UniversityTaizhou317000P.R. China; ^4^ Department of Chemical EngineeringNational Cheng Kung UniversityTainan70101Taiwan; ^5^ Department of Materials Science and EngineeringNational Chiao Tung University300HsinchuTaiwan

**Keywords:** organic photovoltaics, perylene diimides, solution‐processed, nonfullerene acceptor, 3D molecules

## Abstract

**A nonfullerene acceptor based on a 3D tetraperylene diimide** is developed for bulk heterojunction organic photovoltaics. The disruption of perylene diimide planarity with a 3D framework suppresses the self‐aggregation of perylene diimide and inhibits excimer formation. From planar monoperylene diimide to 3D tetraperylene diimide, a significant improvement of power conversion efficiency from 0.63% to 3.54% can be achieved.

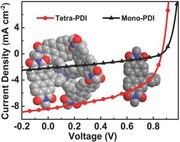

Organic photovoltaics (OPVs) made from electron donor–acceptor (D–A) bulk heterojunction (BHJ) based devices have attracted extensive attention due to their merits of light‐weight, low‐cost, flexibility, and solution processability. Fullerene derivatives with unique phase and multidimensional charge‐transporting capabilities have played a central role as electron acceptor in BHJ devices leading to ≈11% power conversion efficiency (PCE).^1^, ^2^ In spite of the widespread usage of fullerene acceptors, they do suffer from drawbacks including weak absorption of visible light, poor chemical and electronic tunability, and high production cost. To expand the horizon of OPVs, it is highly desirable to develop nonfullerene acceptors that are able to retain the advantages of fullerenes but without the abovementioned deficiencies. Inspired by the rapid development of electron‐transporting materials, some molecular nonfullerene acceptors^3^ such as perylene diimides (PDIs),^4^, ^5^, ^6^ naphthalene diimide,^7^ fluoranthene‐fused imide,^8^ decacyclene triimdes,^9^ tetraazabenzodifluoranthene diimides,^10^ benzothiadiazole imide,^11^ diketopyrrolopyrrole (DPP),^12^ dicynodistyrylbenzene,^13^ and azadipyrromethene complexes^14^ have all been developed for OPVs. Among these, PDI is the most commonly used basic unit for OPV nonfullerene acceptors.^4^, ^5^, ^6^ By using a bay‐linked nonplanar di‐PDI as electron acceptor, Jen and others have achieved PCE as high as ≈6%, which is among the highest values reported for small molecule nonfullerene OPVs.[[qv: 5a–c]] Nonfullerene acceptors with nonplanar structures have in general shown superior performance than those of the planar analogs in solution‐processed OPVs.^5^, ^6^, ^8^,[[qv: 10a]],^12^, ^13^, ^14^ The molecular geometries of the PDI nonfullerene acceptors evolve from planar monomer,^4^ twisted dimer^5^ to quasi‐3D and 3D structures^6^ (Figure S1, Supporting Information), in order to suppress the self‐aggregation of planar molecules for facilitating nanoscale phase domain formation. More importantly, it enhances multidimensional charge separation (with donor) and transport.[[qv: 5g–i,]]^15^, ^16^

In this work, we report a synthetic strategy to effectively transform planar subunits into a 3D nonfullerene acceptor with a compact tetrahedron core, of which four subunits are interlocked to reinforce structural rigidity of the molecule. The resulting molecule shows interesting correlations between its molecular dimensionality, material properties, and device performance. By coupling tetraphenyl‐silane with four planar PDIs to build a 3D nonfullerene acceptor (denoted as tetra‐PDI, **Figure** **1**a), it disrupts the PDI planarity with an interlocked 3D geometry that can suppress the PDI aggregation and facilitate excitation energy transfer between PDI subunits. More importantly, tetra‐PDI possesses significantly improved charge‐transporting properties than those of planar mono‐PDI in both neat films and BHJ films. These combined attributes result in an improved PCE (3.54%) in a proof‐of‐concept OPV device based on tetra‐PDI, which is about five times higher than that of device using planar mono‐PDI subunit.

**Figure 1:**
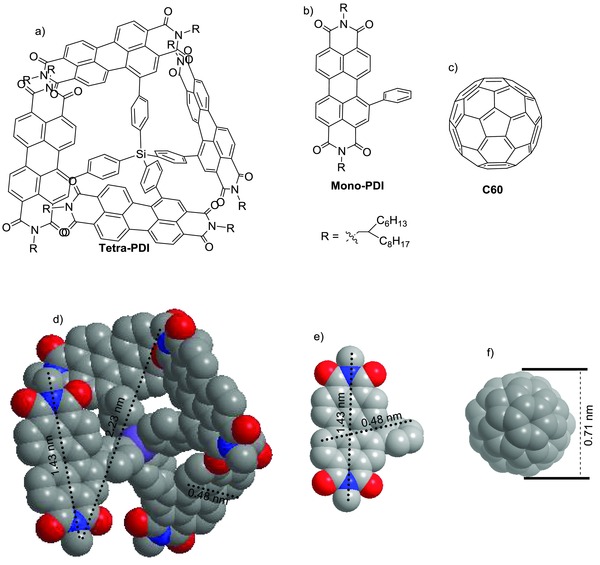
DFT optimized geometries of a,d) tetra‐PDI and b,e) mono‐PDI shown by models of ChemDraw (top row) and space filling (bottom row). c,f) C_60_ for comparison.

Density functional theory (DFT) calculations were carried out initially to study the molecular structure of 3D tetra‐PDI (Figure 1a,d) and planar PDI subunit (mono‐PDI, Figure 1b,e). Figure 1a shows that tetraphenylsilane possesses a 3D geometry that holds PDI units in four directions according to the sp^3^ hybrid of silicon core. Although each PDI unit and phenyl group in tetra‐PDI are covalently linked by rotatable single bonds, the four PDI units on the compact Si core has a 3D interlocked geometry, which is nonrotatable and differs from the previously reported nonplanar molecules.^5^, ^6^

Tetra‐PDI gathers four PDI units into a crowded space (probably the smallest possible) that will benefit the energy transfer and electron communication among PDI units. Actually, our initial attempt to install bulky PDI units onto tetra‐Ph‐methane failed due to the smaller core size of tetra‐Ph‐methane (C−C bond length ≈ 1.5 Å) compared to tetra‐Ph‐silane (C−Si bond length ≈ 1.9 Å) (Figure S2, Supporting Information). The four PDI π‐planes (with a length of 14.3 Å and a width of 4.8 Å) in tetra‐PDI expose to the outside sphere of tetra‐Ph‐silane. DFT calculations indicate that tetra‐PDI can be seen as a molecule mimics fullerene derivatives in terms of π‐surface exposure and electron affinity (C_60_ with a diameter of 7.1 Å, Figure 1c,f) if alkyl chains are not taken into account. Structurally, the 3D‐like tetra‐PDI possesses certain advantages over the planar ones that can allow charge separation and transport from different directions^15^, ^16^ and can inhibit the commonly observed excimer formation at the D–A interfaces.^17^, ^18^

Tetra‐PDI and mono‐PDI were synthesized via the Pd(dppf)­Cl_2_‐catalyzed Suzuki coupling reactions between *N*,*N’*‐di(2‐hexyldecyl)‐1‐bromo‐3,4:9,10‐perylene diimide (**1**) and tetrakis(4‐(4,4,5,5‐tetramethyl‐1,3,2‐dioxaborolan‐2‐yl)phenyl)­silane (**2**) and phenylboronic acid, respectively (**Scheme** **1**). The Suzuki coupling reaction between **1** and **2** afforded tetra‐PDI in 70% yield. In particular, Pd(dppf)Cl_2_ is found to have a much higher catalytic activity than the traditionally used Pd(PPh_3_)_4_, and tetrahydrofuran (THF) is a better solvent than toluene for the above Suzuki coupling reactions, especially for synthesizing highly steric‐hindered tetra‐PDI. Both tetra‐PDI and mono‐PDI are readily soluble in common organic solvents such as dichloromethane, THF and dichlorobenzene at room temperature due to the presence of solubilizing *N*‐substituted alkyl chains. The target molecules were fully characterized by ^1^H and ^13^C NMR, matrix‐assisted laser desorption ionization time of flight (MALDI–TOF) mass spectroscopy (MS). Interestingly, the results from ^1^H and ^13^C NMR spectra indicate that the chemical environments of the two *N*‐alkyl chains on tetra‐PDI are very different from those of the mono‐PDI subunits and previously reported bay‐substituted PDIs.[[qv: 5b–g]],^6^ As shown in **Figures** **2**a, S3, and S4, Supporting Information, the –NCH_2_– groups on mono‐PDI exhibit a set of *dd* peaks around 4.08 ppm (^1^H NMR), while the chemical shifts of the –NCH_2_– groups on tetra‐PDI are equally divided into two sections, one at 4.14 ppm and the other at 3.55 ppm. ^1^H NMR signals of the –(CH_2_)*_n_*– and –CH_3_ groups on tetra‐PDI also exhibit substantial splits. Splits were also abserved from the ^13^C NMR signals of tetra‐PDI (insertion on the top left of Figure 2a and Figure S4, Supporting Information). Half of the alkyl hydrogen and carbon atoms on tetra‐PDI shift to higher fields due to the magnetic field shielding of π‐electrons on the neighboring PDI π‐rings. These observations indicate that the surrounding of the PDI units on tetra‐PDI is compact, interlocked, and nonrotatable, suggesting tetra‐PDI possesses a 3D yet rigid architecture that to some extent like fullerenes.

**Scheme 1:**
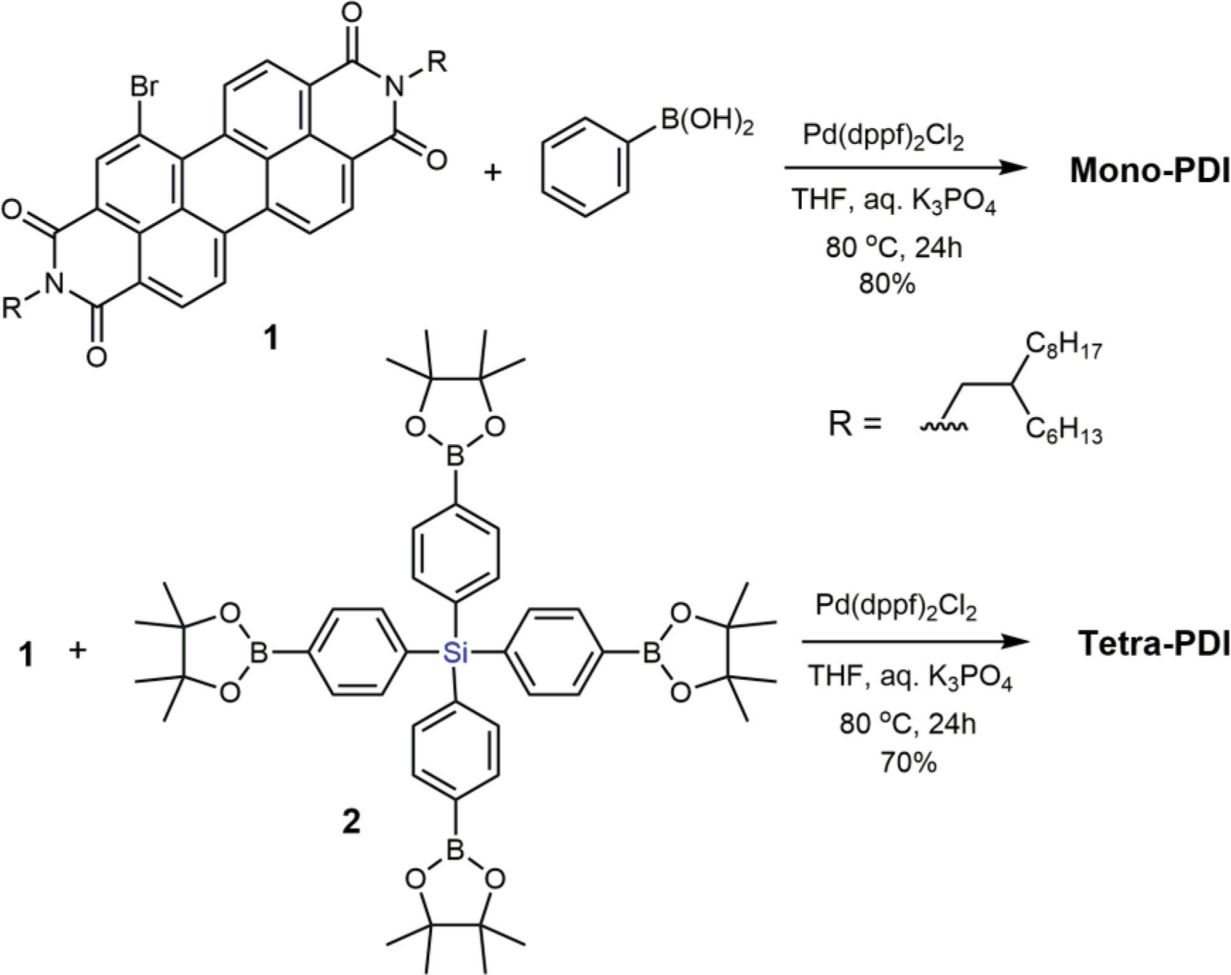
Synthesis of mono‐PDI and tetra‐PDI.

**Figure 2:**
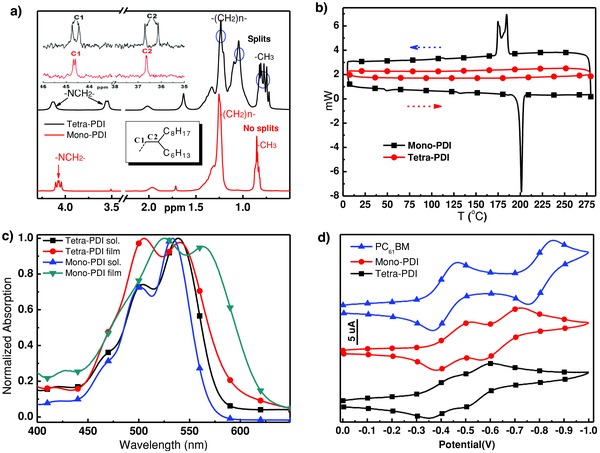
a) ^1^H NMR signal of *N*‐alkyl chain of tetra‐PDI and mono‐PDI (The insertion on top left is ^13^C NMR for C1 and C2); b) DSC curves of tetra‐PDI and mono‐PDI; c) UV–vis absorption spectra of tetra‐PDI and mono‐PDI in solution and solid films; d) CV curves for tetra‐PDI, mono‐PDI, and PC_61_BM in CH_2_Cl_2_ solutions.

Aside from the molecular structure study, to gain information of intermolecular interactions and crystallinity for both compounds, differential scanning calorimetry (DSC) was used to investigate their thermal behaviors. As shown by Figure 2b, the DSC scan of mono‐PDI from 5 to 280 °C exhibits both intense crystalline melting and recrystallization peaks at 200 and ≈180 °C, respectively, implying mono‐PDI has high crystallinity due to its structural planarity. However, under the similar DSC scans, neither melting nor recrystallization peaks could be observed for tetra‐PDI (Figure 2b), suggesting tetra‐PDI is noncrystalline in nature.^19^, ^20^ By introducing a 3D silane core, the overwhelming aggregation of planar PDI^21^ could be suppressed. More importantly, tetra‐PDI exhibits inherent advantages of having good intramolecular interactions among PDI subunits, as evident by the above DFT calculation and NMR analyses.

Optical and electronchemical properties were subsequently investigated. Figure 2c shows the normalized UV–vis absorption spectra of tetra‐PDI and mono‐PDI in CH_2_Cl_2_ solution (0.8 ×10 ^−7^ m), as well as in thin solid films. Tetra‐PDI and mono‐PDI in CH_2_Cl_2_ solution exhibit absorption peaks at 539 and 536 nm, respectively, with the maximum extinction coefficients of 1.4 × 10^5^ and 9 × 10^4^ m ^−1^ cm^−1^, accordingly. Compared with the absorption in solution, the thin film of tetra‐PDI shows a similar profile with a very small redshift of absorption peak from 539 to 541 nm. However, mono‐PDI shows absorption peaks at 536 nm for solution while 563 nm for film. The large redshift of film absorption (27 nm) indicates that mono‐PDI has a strong tendency to aggregate. The UV–vis results of tetra‐PDI and mono‐PDI support the observations from DSC. Time‐resolved fluorescence spectroscopy was also applied to test the photoluminescence (PL) lifetime of tetra‐PDI and mono‐PDI in both solutions and films (Figure S5, Supporting Information). It was found that tetra‐PDI had a faster PL decay (*t* = 6.38 ns in solution and 10.96 ns in film) than mono‐PDI (*t* = 8.60 ns in solution and 15.07 ns in film). The shorter PL lifetime of tetra‐PDI may ascribe to the faster intramolecular excitation energy transfer between PDI units of 3D tetra‐PDI, which will favor charge separation and transport in BHJ.^18^

The electrochemical properties of tetra‐PDI and mono‐PDI were checked by cyclic voltammetry (CV) in CH_2_Cl_2_ solution. Both PDIs undergo two quasi‐reversible reduction events and neither shows any oxidative activity in the scan range (Figure 2d). The lowest unoccupied molecular orbital (LUMO) levels were estimated from the *E_1_*_/2_ values in solution, using the value of −5.1 eV for Fc/Fc^+^.^22^ The LUMOs for tetra‐PDI and mono‐PDI were −4.00 and −3.98 eV, respectively. The highest occupied molecular orbital levels of tetra‐PDI and mono‐PDI are estimated to be −5.97 and −5.98 eV, respectively, by subtracting the LUMO levels from the optical bandgaps (1.97 eV for tetra‐PDI and 2.00 eV for mono‐PDI). The LUMO levels of tetra‐PDI and mono‐PDI are very close to that of PC_61_BM (Figure 2d), indicating both PDI derivatives are suitable n‐type materials for OPV.

To study the role of tetra‐PDI in BHJ OPVs, the commercially available poly({4,8‐bis[(2‐ethylhexyl)oxy]benzo[1,2‐b:4,5‐b′]dithiophene‐2,6‐diyl}{3‐fluoro‐2‐[(2‐ethylhexyl)‐carbonyl]‐thieno[3,4‐b]thiophenediyl}) (PBDTT‐F‐TT, structure shown in Figure S6, Supporting Information) was chosen as the donor, which has complementary absorption spectrum and matched frontier energy levels (Figure S6, Supporting Information) with tetra‐PDI. As shown in **Figure** **3**a, PBDTT‐F‐TT with an intense absorption band between 600 and 750 nm complements well with the absorption between 450 and 600 nm for tetra‐PDI. The inverted devices with a configuration of indium tin oxide (ITO)/ZnO/PBDTT‐F‐TT:PDIs/MoO_3_/Ag (Figure S7, Supporting Information) were fabricated with *ortho*‐dichorobenzene (DCB) as the processing solvent to investigate the performance of solution‐processed BHJ OPVs.[[qv: 5a]] After optimizing the blend ratio of the active layer to be 1:1.2 between PBDTT‐F‐TT and tetra‐PDI, the device showed a PCE of 3.21% (with a *V*_OC_ of 0.87 V, a short circuit current (*J*_SC_) of 7.66 mA cm^−2^, and a fill factor (FF) of 0.48) (Figure 3b and **Table** **1**).

**Table 1:** The best and average (in brackets, at least eight devices) device data of OSCs based on PBDTT‐F‐TT:tetra‐PDI and mono‐PDI under the illumination of air‐mass 1.5G, 100 mW cm^−2^

D:Aa)	Additive	*V*_OC_ [V]	*J*_SC_ [mA cm^−2^]	FF	PCE [%]	Cal. *J*_SC_ b) [mA cm^−2^]
PBDTT‐F‐TT:tetra‐PDI	–	0.87 ± 0.00	7.56 ± 0.11	0.48 ± 0.01	3.21(3.13)	7.84
	3% CN	0.86 ± 0.00	8.25 ± 0.13	0.48 ± 0.01	3.54(3.37)	8.65
PBDTT‐F‐TT:mono‐PDI	–	0.85 ± 0.01	2.10 ± 0.10	0.33 ± 0.01	0.63(0.59)	2.31

^a)^The D:A ratio is 1:1.2 (w/w);

^b)^Calculated from EQE. Average values from 12 devices for PBDTT‐F‐TT:tetra‐PDI with or without CN and from eight devices for PBDTT‐F‐TT:mono‐PDI.

**Figure 3:**
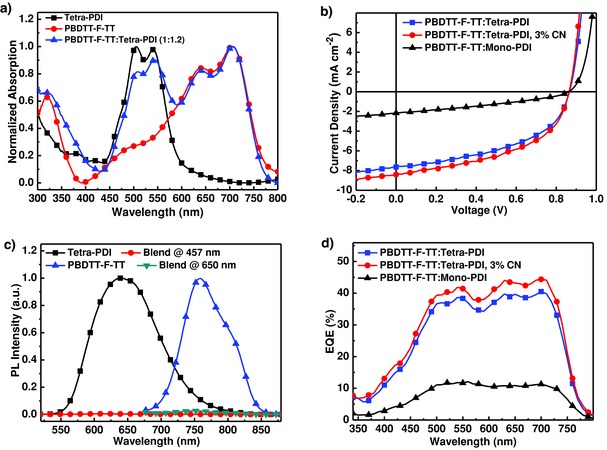
a) UV–vis spectra of neat films of tetra‐PDI and PBDTT‐F‐TT, and their blend film; b) *J*–*V* characteristics of the PBDTT‐F‐TT:tetra‐PDI or mono‐PDI BHJ‐based device with inverted structure; c) Steady PL spectra of tetra‐PDI (excited at 457 nm), PBDTT‐F‐TT (excited at 650 nm), and PBDTT‐F‐TT:tetra‐PDI (1:1.2, w/w) (excited at 457 and 650 nm); d) EQE spectra of PBDTT‐F‐TT:tetra‐PDI with or without CN, and PBDTT‐F‐TT:mono‐PDI.

This was accomplished without applying any posttreatments or additives; meaning tetra‐PDI possesses favorable properties for OPVs. Similarly fabricated and tested PBDTT‐F‐TT:mono‐PDI only gave a low PCE of 0.63%, with a *J*_SC_ = 2.18 mA cm^−2^, a *V*_OC_ = 0.86 V, and a FF = 34%. Although the *V*_OC_ of mono‐PDI solar cell is similar to that of tetra‐PDI, as expected from their similar LUMO energy levels, the photocurrent and PCE of tetra‐PDI‐based devices are almost five times higher than that of mono‐PDI. The rigid 3D architecture of tetra‐PDI lead to more efficient charge separation and transport, which increase the *J*_SC_ and FF. Processing additives such as 1,8‐diiodooctane (DIO) and 1‐chloronaphthalene (CN) have been widely used for nonfullerene‐based OPVs to improve PCEs.[[qv: 4c–f]],[[qv: 5a–h]],^6^, ^7^, ^9^ However, DIO was found to have a negative effect on the performance of tetra‐PDI based devices. Only the addition of 3% CN into PBDTT‐F‐TT:tetra‐PDI led to an improved PCE of 3.54% (*V*_OC_ of 0.86 V, *J*_SC_ of 8.39 mA cm^−2^ and FF of 49%) (Table 1). Figures S8 and S9 and Tables S1 and S2, Supporting Information provide the detailed information of device optimization.

To better understand the underlying mechanisms for these results, PL spectra of neat films of PDIs and PBDTT‐F‐TT and their blends, in conjunction with the incident photon to current conversion efficiency were investigated. By comparing the PL of 1:1.2 blend films with those of neat films, it shows almost complete quenching (>95%) of both the PBDTT‐F‐TT and tetra‐PDI emission, which suggests efficient charge transfer driven by both PBDTT‐F‐TT and tetra‐PDI (Figure 3c). Although efficient PL quenching is also observed in donor and mono‐PDI BHJ (Figure S10, Supporting Information), the external quantum efficiency (EQE) of mono‐PDI BHJ is much lower than that of tetra‐PDI. The EQE spectra and *J*–*V* curves for mono‐PDI and tetra‐PDI BHJs with or without CN additives are shown in Figure 3b,d. The calculated current densities from EQE spectra matched well with those measured values of OPVs^23^ (mono‐PDI, tetra‐PDI, and tetra‐PDI with 3% CN are 2.31, 7.84, and 8.65 mA cm^−2^, respectively (Table 1). Ideally, one would anticipate that efficient BHJ enables photo‐generated charge transfer between donor and acceptor. As shown in Figure 3a,d, the UV–vis and EQE spectra of tetra‐PDI BHJ correlate well with each other indicating charge generation from both PBDTT‐F‐TT and tetra‐PDI. These results confirm the efficient charge photogeneration and photovoltaic properties of 3D tetra‐PDI.

Atomic force microscopy (AFM) was also employed to investigate the surface morphology of the active layers. AFM scans were carried out for 1:1.2 ratio blend films spin‐coated on ITO substrates using the same fabrication conditions for devices. **Figure** **4** shows the AFM images of PBDTT‐F‐TT:mono‐PDI, PBDTT‐F‐TT:tetra‐PDI, and PBDTT‐F‐TT:tetra‐PDI with 3% CN. The corresponding root mean square roughness is 1.90, 1.99, and 2.41 nm, respectively. The surface of PBDTT‐F‐TT:mono‐PDI and PBDTT‐F‐TT:tetra‐PDI are found to be quite smooth and homogenous. This suggests that the difference found in device performance may be mainly due to charge transport in the active layers. The results from space‐charge limited current (SCLC) measurements show that hole mobility of the PBDTT‐F‐TT:tetra‐PDI layer is about five times higher than that of the PBDTT‐F‐TT:mono‐PDI layer. The tetra‐PDI is expected to interact less strongly with the donor polymer due to its nonplaner molecular geometry compared to the planer mono‐PDI. The tetra‐PDI would promote polymer's self‐aggregation in the blend to result in better percolation/phase separation and transport pathways for hole transport compared to the polymer:mono‐PDI blend. This agrees with the PCE trend observed. However, our attempt to measure electron mobility in the PBDTT‐F‐TT:mono‐PDI layer by SCLC method failed due to extremely low electron mobility of mono‐PDI. It can only be measured through the organic field‐effect transistor (OFET) technique. Tetra‐PDI pristine film has one order higher electron mobility (1.4 × 10^−3^ cm^2^ V^−1^ s^−1^) than that of mono‐PDI pristine film (1.2 × 10^−4^ cm^2^ V^−1^ s^−1^). This suggests the introduction of Si‐cored 3D framework improves charge‐transport, which in turn, improves the performance of tetra‐PDI based devices. We noted that previous mono‐PDIs with bay‐substitution (with even smaller atoms like, –F, –Br, and –Cl) exhibited very low electron mobility, ranging from ≈10^−6^ to 10^−4^ cm^2^ V^−1^ s^−1^.^24^ Our tetra‐PDI with a nonplaner, and interlocked 3D structure with four PDI moieties orientating to different directions exhibited multidimensional charge‐transporting capabilities. This 3D geometry was engineered to mimic fullerene's charge‐transporting behaviors, thus tetra‐PDI showed increased electron mobility than that of the planar mono‐PDI. The use of CN additive also contributes to the increased phase separation between PBDTT‐F‐TT and tetra‐PDI, creating additional percolation pathways or interconnected domains for electron transporting in the active layer. This results in more balanced charge transport in the PBDTT‐F‐TT:tetra‐PDI layer for improving *J*_SC_ and PCE (Table S3, Supporting Information).

**Figure 4:**
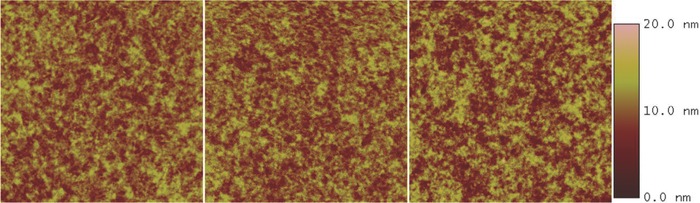
AFM height images of PBDTT‐F‐TT:mono‐PDI (left), PBDTT‐F‐TT:tetra‐PDI (middle), and PBDTT‐F‐TT:tetra‐PDI (right) with 3% CN. Scale: 5 μm × 5 μm.

In summary, we have developed a novel PDI‐based 3D nonfullerene electron acceptor with an interlocked architecture, which originates from the sp^3^ hybrid silane framework. The Si‐cored tetra‐PDI is small and compact to constrain the free rotation of PDI‐subunits. As shown by NMR, DSC, UV–vis, and PL lifetime analyses, this 3D yet interlocked structure can suppress the commonly observed aggregation in planar PDI to facilitate charge transport and energy transfer among PDI subunits. From planar mono‐PDI to 3D tetra‐PDI, improved performance could be achieved for a proof‐of‐concept device without using any posttreatments or additives. This represents an important development for solution‐processed BHJ OPVs, and demonstrates a new strategy for designing 3D electron‐accepting materials with favorable properties for OPV applications.

## Experimental Section

*Synthesis of **1** and **2***: Details for the synthesis of intermediates *N,N′*‐di(2‐hexyldecyl)‐1‐bromo‐3,4:9,10‐perylene diimide (**1**) and tetrakis(4‐(4,4,5,5‐tetramethyl‐1,3,2‐dioxaborolan‐2‐yl)phenyl)silane (**2**) can be found in Scheme S1 and S2, Supporting Information.^24^

*Synthesis of Tetra‐PDI*: Compound **1** (229 mg, 0.25 mmol), compound **2** (50.5 mg, 0.06 mmol), THF (6 mL), and K_3_PO_4_ aqueous solution (1 m, 1 mL) were mixed together in a two‐necked round bottom flask. The mixture was degassed by argon, then 10 mg Pd(dppf)Cl_2_·CH_2_Cl_2_ (0.0125 mmol) was added. The mixture was stirred and refluxed under argon at 80 °C for 24 h. After cooling to room temperature, the mixture was poured into 100 mL NaCl aqueous solution and extracted with CH_2_Cl_2_ several times. The organic layers were combined, dried over MgSO_4_ and filtered. Removal of solvent by rotary evaporator afforded the crude product, which was then purified by silica gel column chromatography using CH_2_Cl_2_ as eluent. Red powder was obtained (159 mg, yield 70%). ^1^H NMR (300 MHz, CDCl_3_, δ): 8.83–8.50 (m, 20H), 8.08 (d, *J* = 7.6 Hz, 8H), 7.92 (d, *J* = 7.5 Hz, 12H), 7.60 (d, *J* = 8.2 Hz, 4H), 4.14 (s, 8H), 3.55 (d, *J* = 6.0 Hz, 8H), 2.21 – 0.41 (m, 248H); ^13^C NMR (126 MHz, CDCl_3_, δ): 163.82, 163.70, 163.18, 162.91, 144.75, 141.49, 138.59, 135.60, 134.92, 134.52, 134.39, 132.98, 131.52, 131.05, 129.78, 128.60, 128.56, 128.04, 127.27, 123.30, 123.41, 122.82, 122.70, 122.41, 121.59, 44.74, 44.45, 36.70, 36.14, 31.87, 31.80, 31.72, 31.53, 31.50, 30.06, 29.83, 29.73, 29.59, 29.47, 29.29, 29.24, 26.49, 26.29, 26.26, 22.64, 22.55, 14.08, 14.01; MALDI–TOF MS *m*/*z*: [M + Na]^+^ calcd for C_248_H_308_N_8_O_16_Si, 3685.32; found, 3687.97.

*Synthesis of Mono‐PDI*: The procedure for the synthesis of mono‐PDI was similar to tetra‐PDI, using THF as solvent, K_3_PO_4_ as base, and Pd(dppf)Cl_2_·CH_2_Cl_2_ as catalyst for the Suzuki coupling of compound **1** (110 mg) with phenylboronic acid (15.9 mg). The target mono‐PDI was obtained (88 mg, yield 80%). ^1^H NMR (300 MHz, CDCl_3_, δ): 8.58–8.31 (m, 5H), 7.99 (d, *J* = 8.3 Hz, 1H), 7.65 (d, *J* = 8.3 Hz, 1H), 7.57–7.38 (m, 5H), 4.08 (dd, *J* = 10.8, 7.3 Hz, 4H), 1.95 (s, 2H), 1.28 (d, *J* = 16.4 Hz, 48H), 0.85 (t, *J* = 4.9 Hz, 12H); ^13^C NMR (126 MHz, CDCl_3_, δ): 163.83, 163.68, 163.53, 142.46, 141.69, 136.03, 134.71, 134.61, 134.31, 132.42, 130.98, 130.82, 130.38, 130.09, 130.02, 128.84, 128.67, 128.41, 128.21, 127.91, 127.37, 123.42, 123.13, 123.03, 122.58, 122.59, 122.17, 44.69, 44.61, 36.66, 36.62, 31.89, 31.87, 31.75, 31.71, 31.66, 30.05, 29.74, 29.59, 29.31, 26.50, 22.66, 14.11; MALDI–TOF MS *m*/*z*: [M + Na]^+^ calcd for C_62_H_78_N_2_O_4_, 915.32; found, 915.89.

*Device Fabrication*: ITO‐coated glass substrates (15 Ω sq^−1^) were cleaned ultrasonically with detergent, deionized water, acetone, and isopropyl alcohol in sequence for 10 min each. Subsequently, they were treated with oxygen plasma for 1 min. A ZnO precursor solution was spin‐coated onto precleaned ITO glass substrates at 4000 rpm for 60 s and then annealed at 200 °C for 1 h in air to complete the thin layer of ZnO (≈30 nm). The ZnO precursor solution was prepared by dissolving zinc acetate dehydrate C_4_H_6_O_4_Zn·2(H_2_O) (99.5%, Merck 1 g) and monoethanolamine (HOCH_2_CH_2_NH_2_, 98% Acros, 0.28 g) in 2‐methoxyethanol (CH_3_OCH_2_CH_2_OH, Aldrich, 98%, 10 mL) under stirring for 8 h for hydrolysis reaction and aging. The active layers (≈85 nm) were obtained by spin‐coating the solution of PBDTT‐F‐TT:PDI acceptors (tetra‐PDI or mono‐PDI) that was filtered with a polytetrafluoroethylene filter (0.45 μm) atop the thin layer of ZnO. The D–A blends were dissolved in DCB with or without 3% DIO or CN and stirred overnight in a nitrogen‐filled glove box. The anode of each device, MoO_3_/Ag (8/150 nm), was thermally evaporated at a pressure of about 10^−7^ torr. The area of each device was 3.14 mm^2^ defined by a shadow mask.

## Supporting information

As a service to our authors and readers, this journal provides supporting information supplied by the authors. Such materials are peer reviewed and may be re‐organized for online delivery, but are not copy‐edited or typeset. Technical support issues arising from supporting information (other than missing files) should be addressed to the authors.

Supplementary
