# Supplementary material for: A Tetraperylene Diimides Based 3D Nonfullerene Acceptor for Efficient Organic Photovoltaics
Source: Adv Sci (Weinh). 2015 Feb 27;2(4):1500014. doi: 10.1002/advs.201500014 (PMC5115352; doi:10.1002/advs.201500014)
Supplement: Supplementary file 1 — Supplementary [file ADVS-2-0e-s001.pdf]

Copyright WILEY-VCH Verlag GmbH & Co. KGaA, 69469 Weinheim, Germany, 2015.

ADVANCED SCIENCE
Open Access

Supporting Information

for *Adv. Sci.*, DOI: 10.1002/advs.201500014

A Tetraperylene Diimides Based 3D Nonfullerene Acceptor for Efficient Organic Photovoltaics

Shi-Yong Liu, Chen-Hao Wu, Chang-Zhi Li, Sheng-Qiang Liu, Kung-Hwa Wei, Hong-Zheng Chen, and Alex K.-Y. Jen**

Copyright WILEY-VCH Verlag GmbH & Co. KGaA, 69469 Weinheim, Germany, 2013.

Supporting Information

A Tetra Perylene Diimde Based Three-Dimensional Non-Fullerene Acceptor for Efficient Organic Photovoltaics

Shi-Yong Liu, Chen-Hao Wu, Chang-Zhi Li, Sheng-Qiang Liu, Kung-Hwa Wei, Hong-Zheng Chen, and Alex K.-Y. Jen**

Contents

1. Instruments, Materials and Experiments..S2

2. **Figure S1** (Structures of planar PDI monomer to 3D tetramer)..S5

3. **Figure S2** (Structural comparison between tetra-Ph-silane and tetra-Ph-methane).............S6

4. **Scheme S1** (Synthesis of intermediate **1**)..S6

5. **Scheme S2** (Synthesis of intermediate **2**)..S6

6. **Figure S3**, **S4** (^{1}H & ^{13}CNMR signals of N-alkyl chains)..S7

7. **Figure S5** (PL decay spetra of Tetra-PDI and Mono-PDI in solutions & films)................S8

8. **Figure S6** (Structure of PBDTT-F-TT and frontier energy level chart).............................S8

9. **Figure S7** (Configuration chart for the OPVs in this study) S9

10. **Figures S8**, **S9**, and **Tables S1**, **S2** (Ratio and additive optimization for OPVs) S9

11. **Figures S10** (Uv-vis and PL of neat & blend films of Mono-PDI and PBDTT-F-TT)...S10

12. **Figures S11, S12** and **Table S3**(OFET and SCLC curves and values) S11

13. **Figures S13-S20** (^{1}H & ^{13}C NMR and MALDI-TOF MS spectra)................................S12

14. **References**.... ....S15

Instruments, Materials and Experiments.

All ^1H and ^{13}C NMR spectra were obtained in chloroform-*d*, with Bruker 300, and Bruker Avance DRX-499. ^{13}C NMR (126 MHz) spectra were measured with a proton-decoupling pulse program. Chemical shifts for ^1H and ^{13}C NMR were referenced to residual signals from $CDCl_3$ (^1H NMR δ =7.26 ppm and ^{13}C NMR δ =77.23 ppm). Matrix-assisted laser desorption-ionizationtime of flight mass spectrometry (MALDI-TOF MS) was performed on a Bruker Autoflex II. Samples were prepared by diluting the molecules in CH_2Cl_2 with the matrix. Differential scanning calorimetry (DSC) were measured on a WCT-2 thermal balance. UV−vis spectra were recorded with a Jasco V-670. PL spectra were measured on Fluoroskan Ascent FL. Time-resolved PL spectra were test on FluoTime 100 Fluorescence Lifetime Spectrometer, exicited by laser wavelength of 470 nm. Cyclic voltammetry (CV) was done on a CHI 660C electrochemical workstation with Pt disk, Pt plate, and standard 10 calomel electrode (SCE) as working electrode, counter electrode, and reference electrode, respectively, in a 0.1 mol L^{-1} tetrabutylammonium hexafluorophosphate (Bu_4NPF_6) CH_2Cl_2 solution. Theoretical calculations based on density functional methods have been performed for Tetra-PDI and Mono-PDI with Gaussian09 program. Becke's three-parameter gradient-corrected functional (B3LYP) with 6-31G(d,p) basis was used to optimize the geometry.[S1] The current-voltage (*J-V*) curves were measured with Keithley 2400 measurement source units at room temperature in air. The photocurrent was measured under a calibrated solar simulator (Abet 300 W) at 100 mW cm^{-2}.

CV was tested on a CHI 660C electrochemical workstation with Pt disk, Pt plate, and standard 10 calomel electrode (SCE) as working electrode, counter electrode, and reference electrode, respectively, in a 0.1 mol L^{-1} tetrabutylammonium hexafluorophosphate (Bu_4NPF_6) CH_2Cl_2 solution.

OPV device characterizations: The J-V characteristics of devices under an AM 1.5 G illumination of 100 mW cm^{-2} were measured by using a Keithley 2400 source-measure unit in a nitrogen-filled glove box. Devices were measured without any shadow masking. The AM 1.5 G illumination was simulated by using an Oriel 300 W Solar Simulator and calibrated by using a silicon photodiode with a protective KG5 filter calibrated by the National Renewable Energy Laboratory (NREL).

The EQE system uses a lock-in amplifier (Stanford Research Systems SR830) to record the short circuit current under chopped monochromatic light.

AFM images were obtained by a NanoScope IIIa (Digital instrument Inc.) operating in the tapping mode.

PBDTT-F-TT is commercially available from 1-Material, Inc as trade name of PCE-10. Unless otherwise specified, chemicals and solvents were purchased from Aldrich.

OFET Device Fabrication and Testing. Bottom-gate bottom-contact devices were fabricated in a nitrogen glovebox by spin-coating 5 mg/mL DCB semiconductor solutions filtered through 0.45 μm PTFE filters with thermally evaporated silver source/drain electrodes (~120 nm thick) on a benzocyclobutene (BCB) coated silicon substrate (with 300 nm silicon oxide on top). The BCB dielectric layer was spin coated at 4000 rpm and baked at 250 °C for overnight. The active layer was spin coated at 1000 rpm and baked at 110°C for 10 min. Last, the device structure was completed by thermal deposition of the Ag gate electrode. All three PDI-based devices were tested under nitrogen atmosphere.

SCLC Mobility Measurements: Space charge-limited currents were tested in electron-only devices with a configuration of ITO/ZnO/PBDTT-F-TT:PDI acceptors/Ca (20 nm)/Al (130 nm) and hole-only devices with a configuration of ITO/PEDOT:PSS/PBDTT-F-TT:PDI acceptor/MoO_3/Ag. The devices were prepared using the above-mentioned procedure. In hole-only deices, PEDOT:PSS (Clevios_ P VP AI 4083) was spin-coated on ITO substrates at

5000 rpm for 30 sec and then annealed at 140 ℃ for 10 min. The mobilities were determined by fitting the dark current according to the following equation:

$$J(V) = \frac{9V^2}{8L^3}\varepsilon_0\varepsilon_r\mu_0 e^{\left(0.89\gamma\sqrt{V/L}\right)}$$

Where J is the dark current density (mA cm^{-2}), μ_0 is the zero-field mobility (cm^2/V sec), ε_0 is the permittivity of free space ($88.54*10^{-12}$ mA sec/V sec), ε_r is the relative permittivity of the material (3), V is the effectie voltage ($V=V_{Applied}-V_{Built\text{-}in}-V_{series\ resistane}$), and L is the thickness of the active layer.

Synthesis of Intermediates:

Scheme S1 shows the synthetic route of intermediate compound 1 according to the reported procedure.[S2-S4] Scheme S2 shows the synthetic route of intermediate compound **2**. Based on the reported procedure with slight modifications,[S5] detailed procedures are as follows. A solution of 1,4-dibromobenzene (11.8 g, 50 mmol) in anhydrous ether (125 mL) was stirred at –10°C under argon and treated dropwise with a solution of butyllithium (20 mL, 2.5 M in hexane, 50 mmol). The resulting mixture was kept at -10°C for 15 min, and then $SiCl_4$ (1.43 mL, 12.5 mmol) was added dropwise. The mixture was stirred at -10°C for 30 min and at 25°C for 12 h. Then 1 M aqueous HCl was added, and the resulting mixture was extracted with ether. The combined extracts were washed with H_2O and brine, dried over $MgSO_4$, and filtered. Volatiles were removed by evaporation under reduced pressure, and the residue was recrystallized twice from ethanol to afford colorless crystal of tetrakis(4-bromophenyl)silane. ^{1}H NMR (300 MHz, $CDCl_3$) δ 7.62 –7.51 (d, J = 8.4, 8H), 7.42 –7.32 (d, J = 8.4, 8H).

A solution of the above obtained tetrakis(4-bromophenyl)silane (1.3 mg, 2.00 mmol) in anhydrous THF (120 mL) was stirred at -78°C under argon and treated dropwise with a solution of butyllithium (6.40 mL, 2.5 M in hexane, 16 mmol). The resulting mixture was kept at -78°C for 30 min, and then 2-isopropoxy-4,4,5,5-tetramethyl-1,3,2-dioxaborolane (32

mmol) was added dropwise. The mixture was stirred overnight while the temperature was allowed to rise to 25°C. Then 100 mL water was added, and the resulting mixture was extracted with ether. The combined extracts were washed brine, dried over $MgSO_4$, and filtered. Volatiles were removed by evaporation under reduced pressure, and the residue was recrystallized twice from ethyl acetate/ethanol to afford white solid compound **2**. ^{1}H NMR (300 MHz, $CDCl_3$) δ 7.80 (dd, *J* = 8.0, 2.8 Hz, 8H), 7.61 –7.52 (d, 8H), 1.36 (s, 48H).

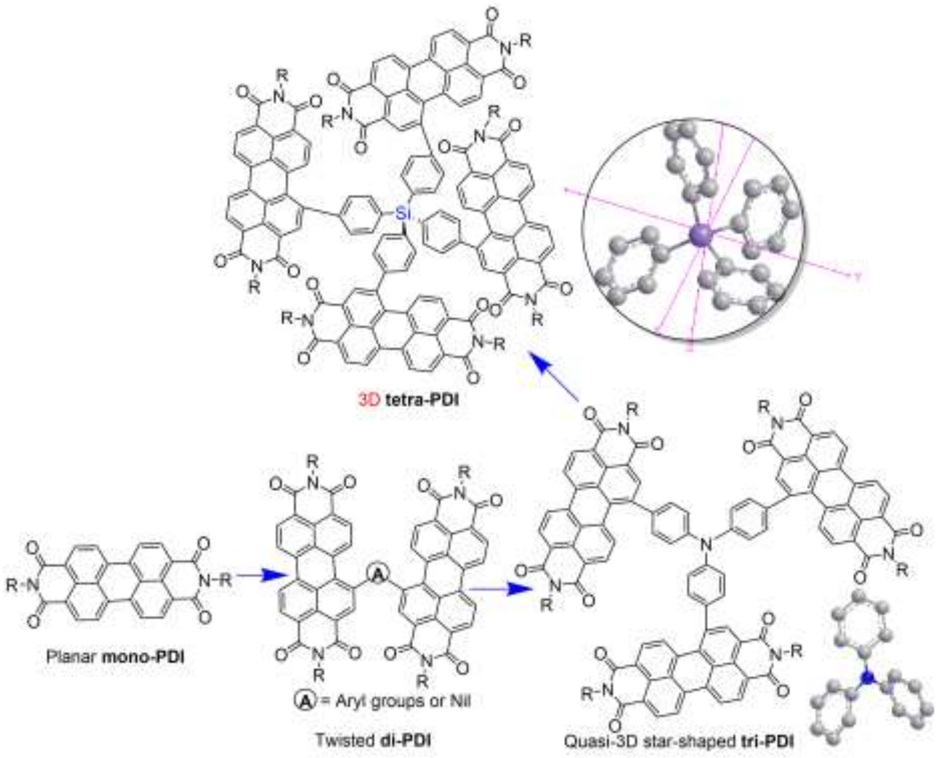

Figure S1. PDI: from planar monomer,[4] twist dimer,[5] pseudo-3D trimer to 3D tetramer.[6]

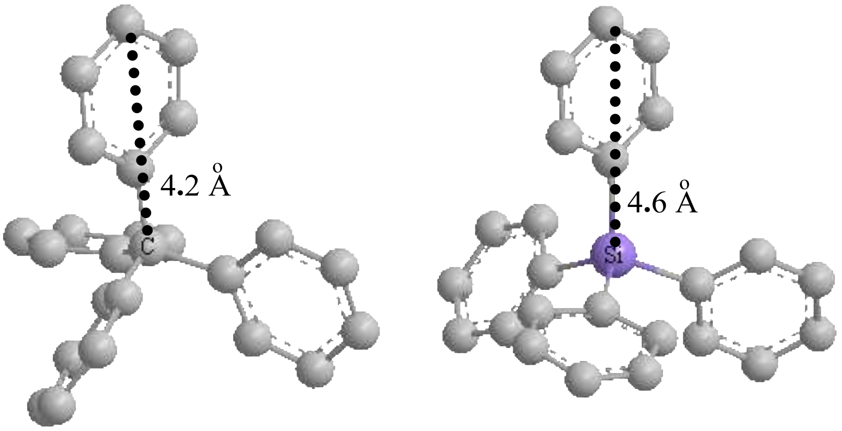

Figure S2. Tetra-Ph-silane has a much larger volume than tetra-Ph-methane, because of $V = 4/3\pi R^3$, $V_{tetra\text{-}Ph\text{-}methane}/V_{tetra\text{-}Ph\text{-}silane} = (4.6/4.2)^3 = 1.31$.

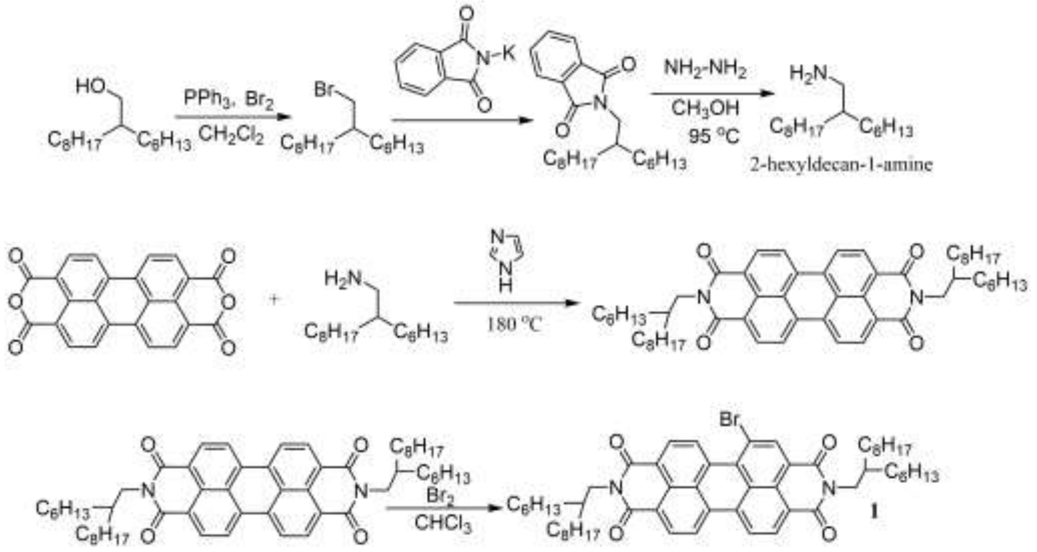

Scheme S1. Synthesis of **1**.[S2-S4]

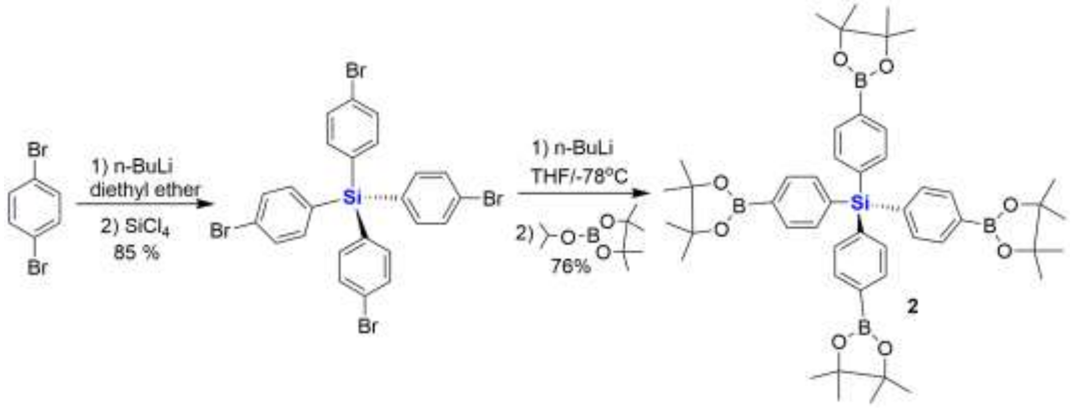

Scheme S2. Synthesis of **2**.[S5]

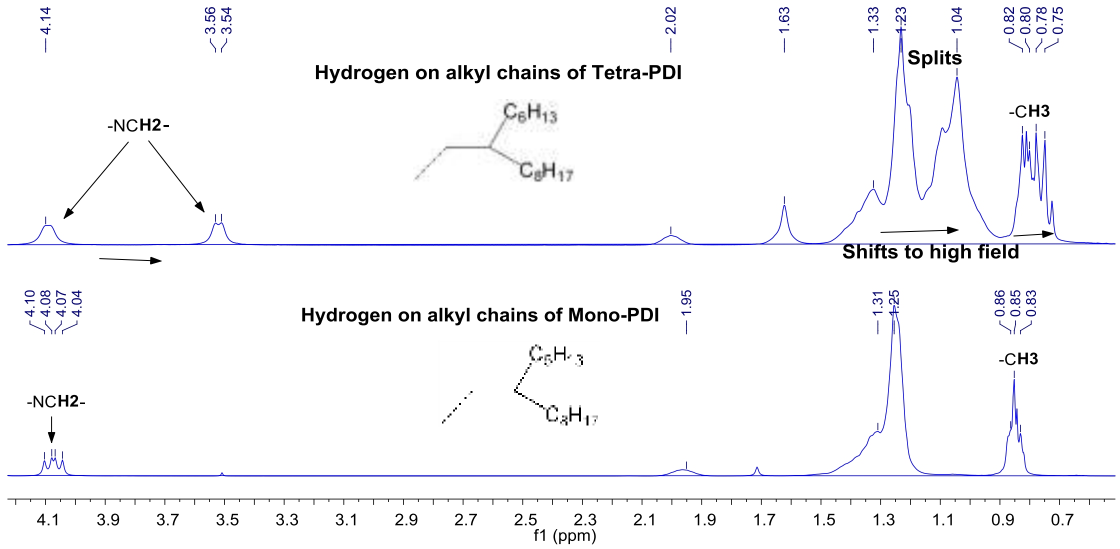

Figure S3. ^{1}H NMR signals of N-alkyl chains.

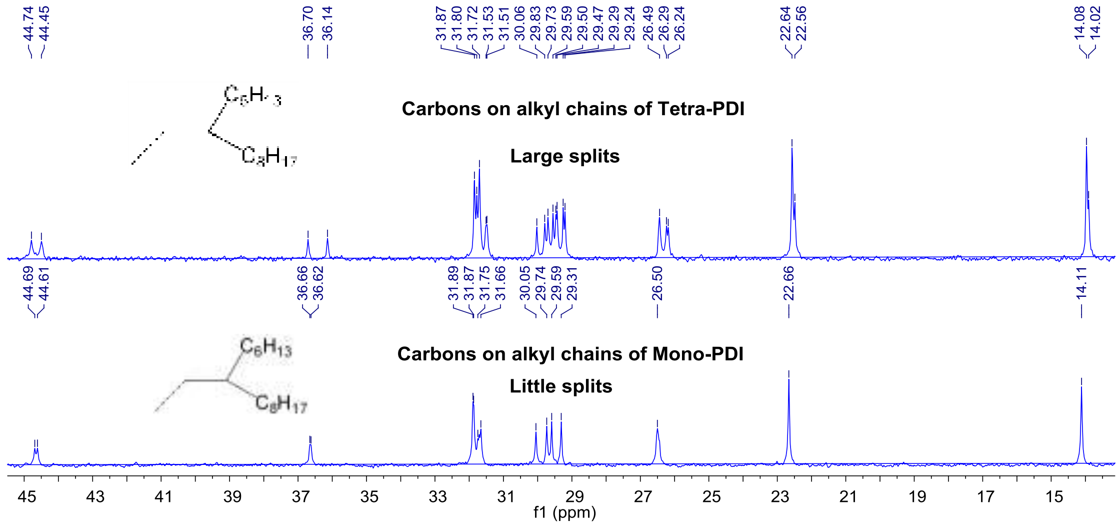

Figure S4. ^{13}C NMR signals of N-alkyl chains.

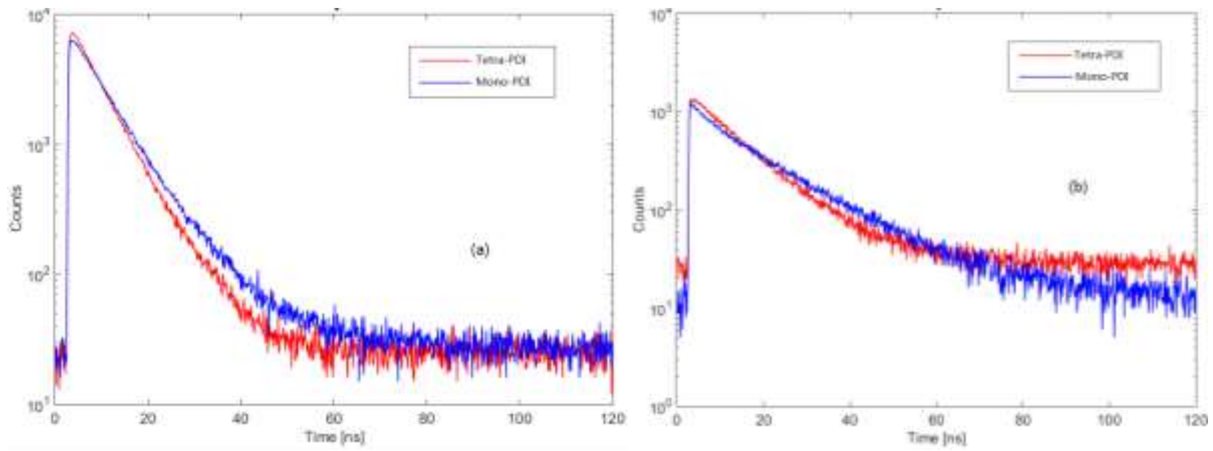

Figure S5. Time-resolved fluorescence spectra of Tetra-PDI and Mono-PDI (a) in CH_2Cl_2 solution and (b) in film, exicited by laser wavelength of 470 nm.

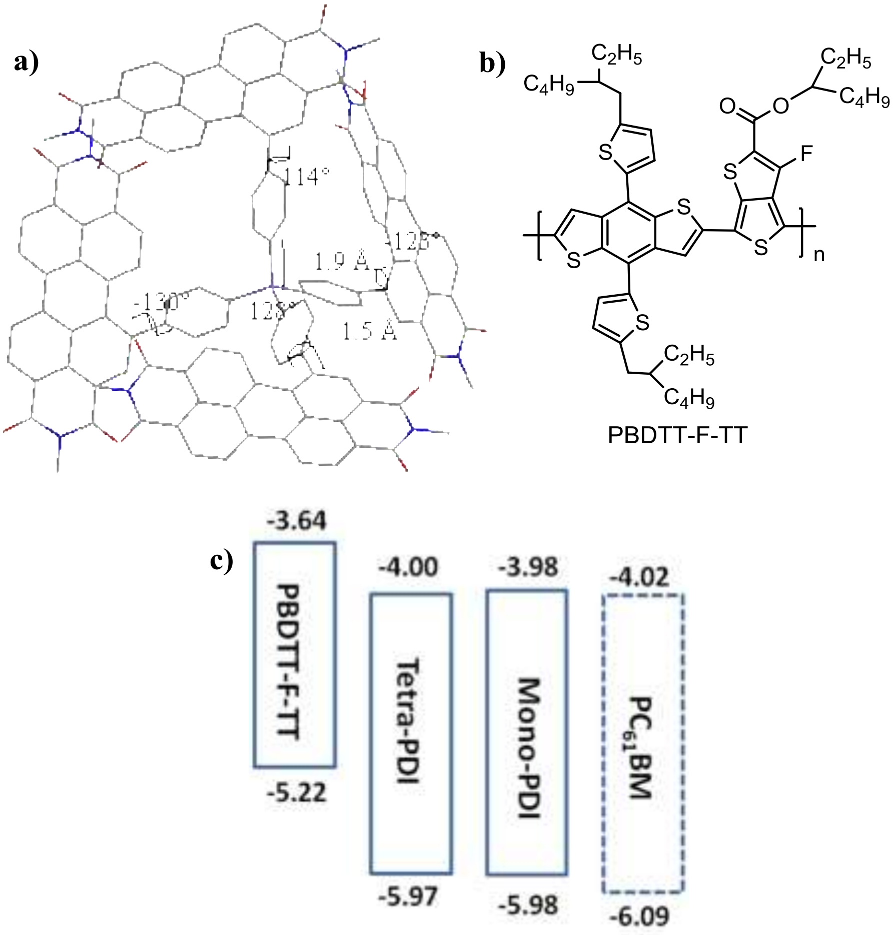

Figure S6. (a) Optimized geometry of Tetra-PDI obtained by DFT shown as cylindrical model; (b) Structure of PBDTT-F-TT; (c) Frontier energy levels of PBDTT-F-TT, Tetra-PDI, Mono-PDI and $PC_{61}BM$.

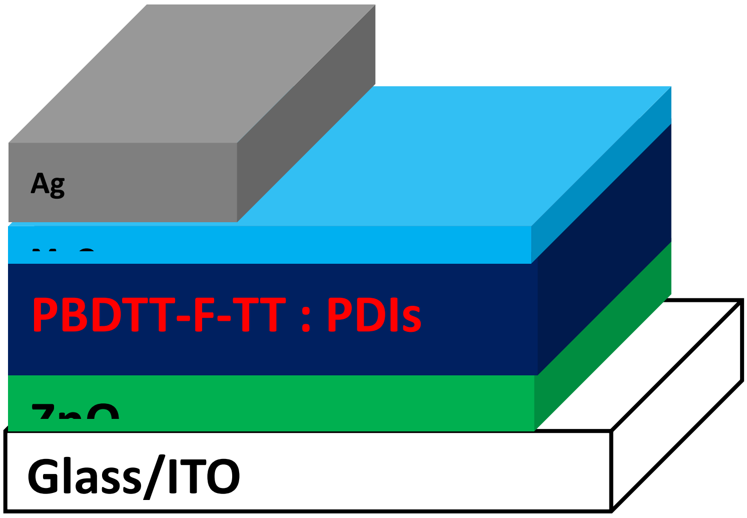

Figure S7. Configuration of invert device for OPVs in this study.

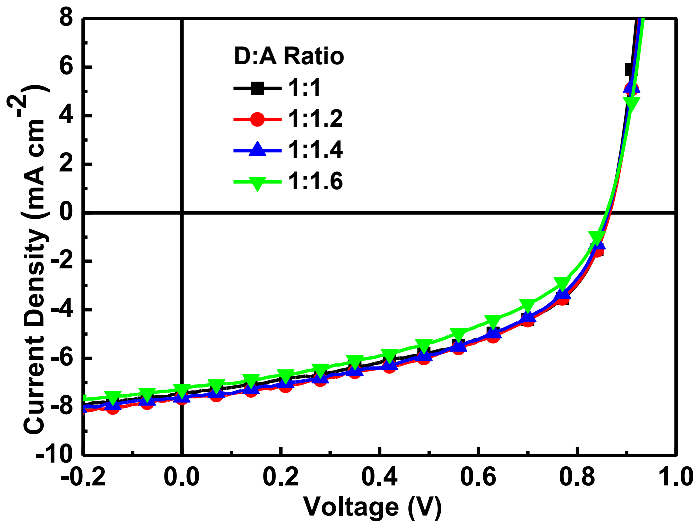

Figure S8. J-V curves of PBDTT-F-TT:Tetra-PDI with various D:A ratios.

Table S1. Effects of D:A ration on the OPV performance of PBDTT-F-TT:Tetra-PDI.

D:A	V_{OC} [V]	J_{SC} [mA cm^{-2}]	FF	PCE [%]
1:1	0.86	7.51	0.48	3.15
1:1.2	0.87	7.66	0.48	3.21
1:1.4	0.86	7.61	0.48	3.14
1:1.6	0.86	7.25	0.45	2.80

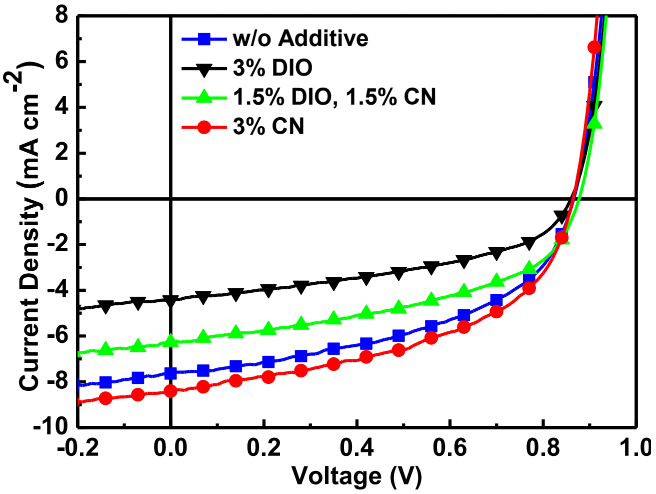

Figure S9. J-V curves of PBDTT-F-TT:Tetra-PDI with DIO and CN additives

Table S2. Effects of additives on the OPV performance of PBDTT-F-TT:Tetra-PDI

Additive	V_{OC} [V]	J_{SC} [mA cm^{-2}]	FF	PCE [%]
No	0.87	7.66	0.48	3.21
3% DIO	0.86	4.42	0.44	1.68
1.5% DIO, 1.5% CN	0.88	6.27	0.47	2.58
3% CN	0.86	8.42	0.49	3.54

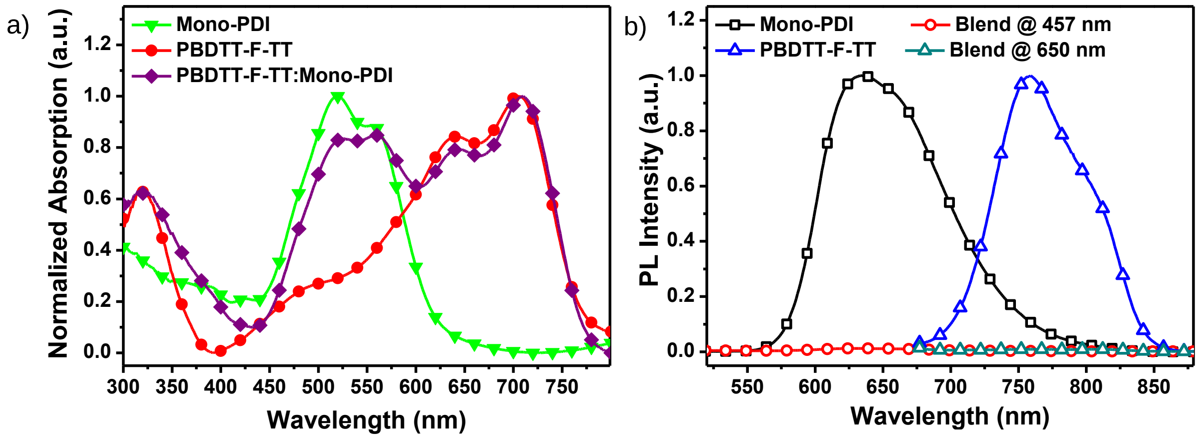

Figure S10. a) UV-vis spectra of neat films of Mono-PDI and PBDTT-F-TT, and their blend film; b) Steady PL spectra of Mono-PDI (excited at 457 nm), PBDTT-F-TT (excited at 650 nm), and PBDTT-F-TT:Mono-PDI (1:1.2, w/w) (excited at 457 and 650 nm).

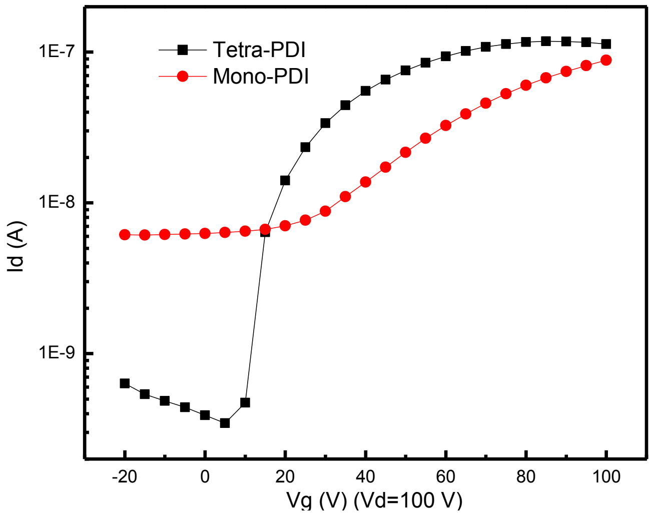

Figure S11. FET characteristic of the Tetra-PDI film.

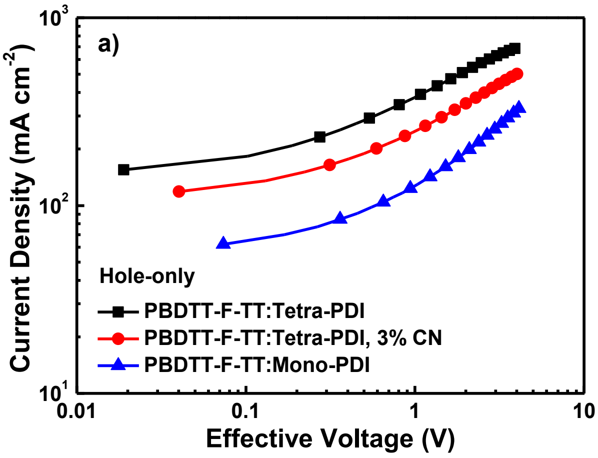

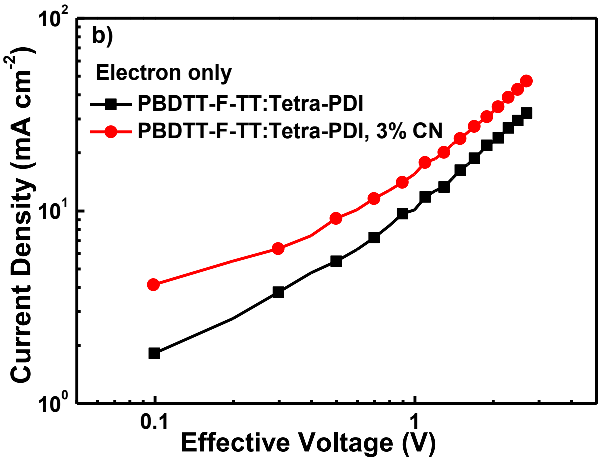

Figure S12. J-V curves for a) hole-only and b) eletron-only devices.

Table S3. Hole and electron mobilities for PBDTT-F-TT:Mono-PDI, PBDTT-F-TT:Tetra-PDI, and PBDTT-F-TT:Tetra-PDI, 3% CN devices

D:A	μ_h [cm^2 V^{-1}S^{-1}]	μ_e [cm^2 V^{-1}S^{-1}]
PBDTT-F-TT:Mono-PDI	6.86×10^{-4}	-
PBDTT-F-TT:Tetra-PDI	3.47×10^{-3}	6.02×10^{-5}
PBDTT-F-TT:Tetra-PDI, 3% CN	1.88×10^{-3}	8.66×10^{-5}

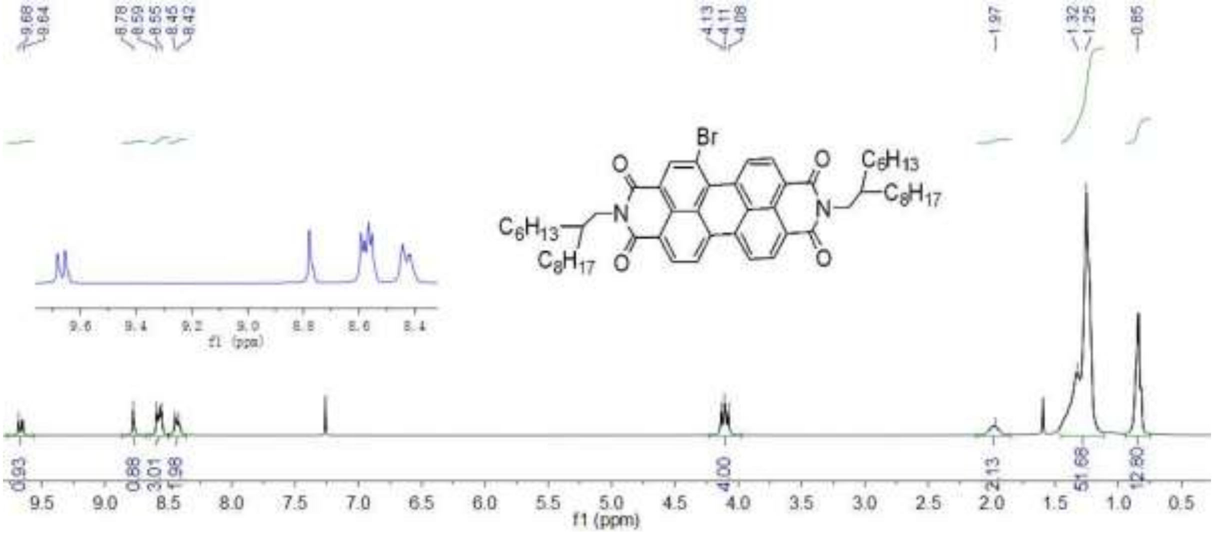

Figure S13.^{1}H NMR of intermediate 1.

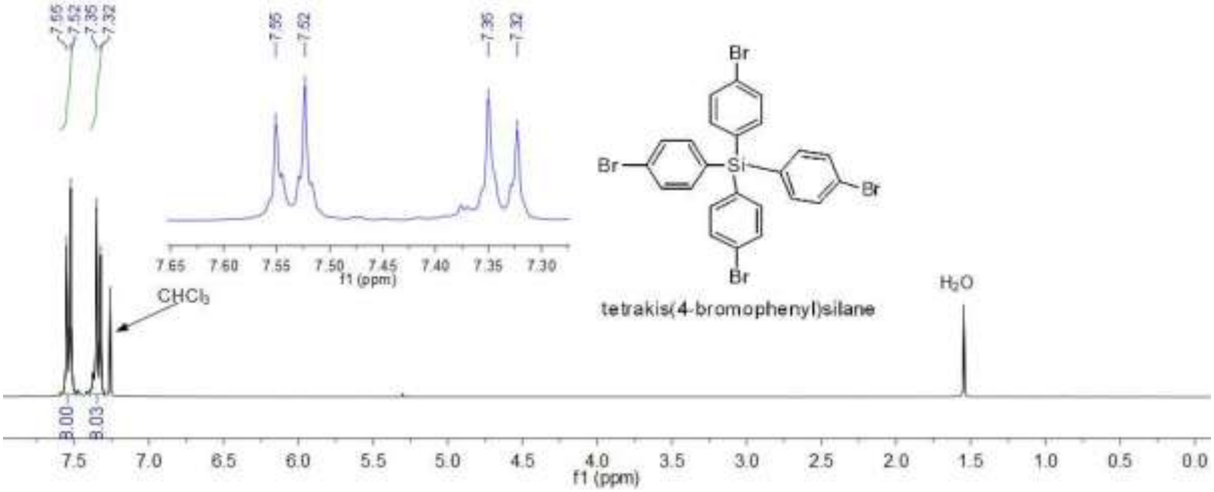

Figure S14. ¹H NMR of tetrakis(4-bromophenyl)silane.

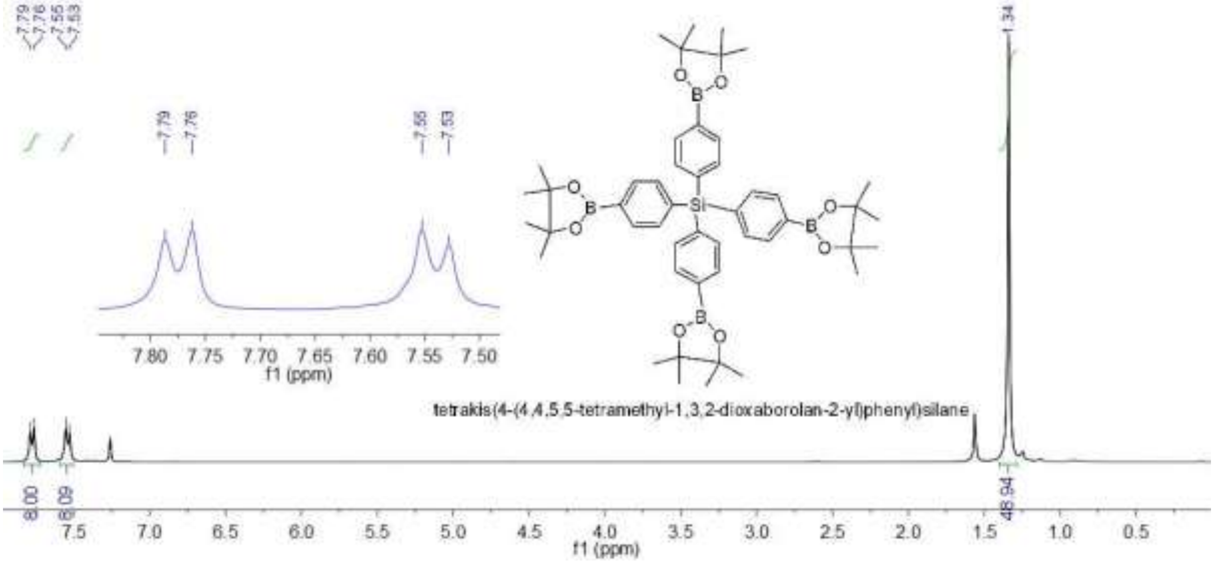

Figure S15. ¹H NMR of intermediate 2.

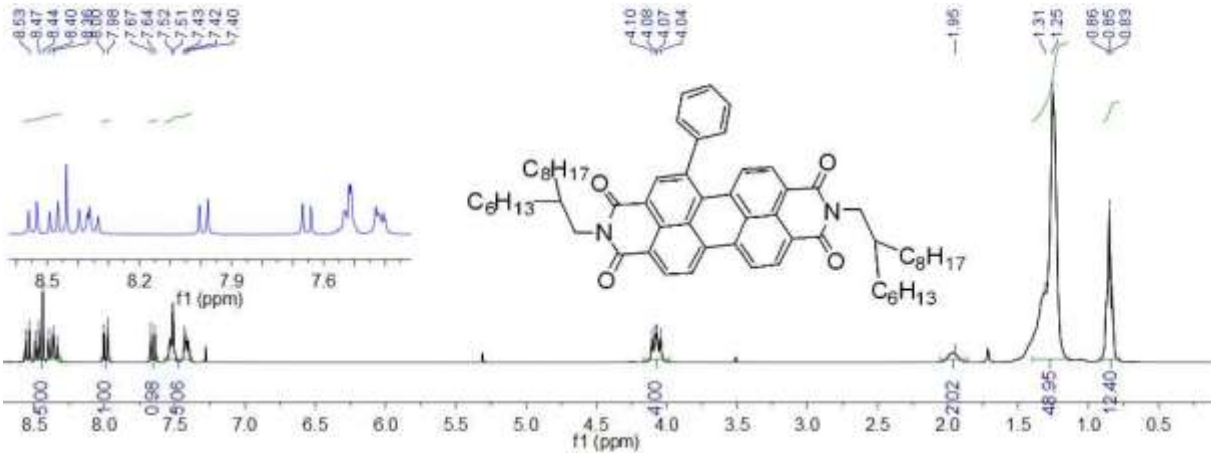

Figure S16. ^{1}H NMR of Mono-PDI.

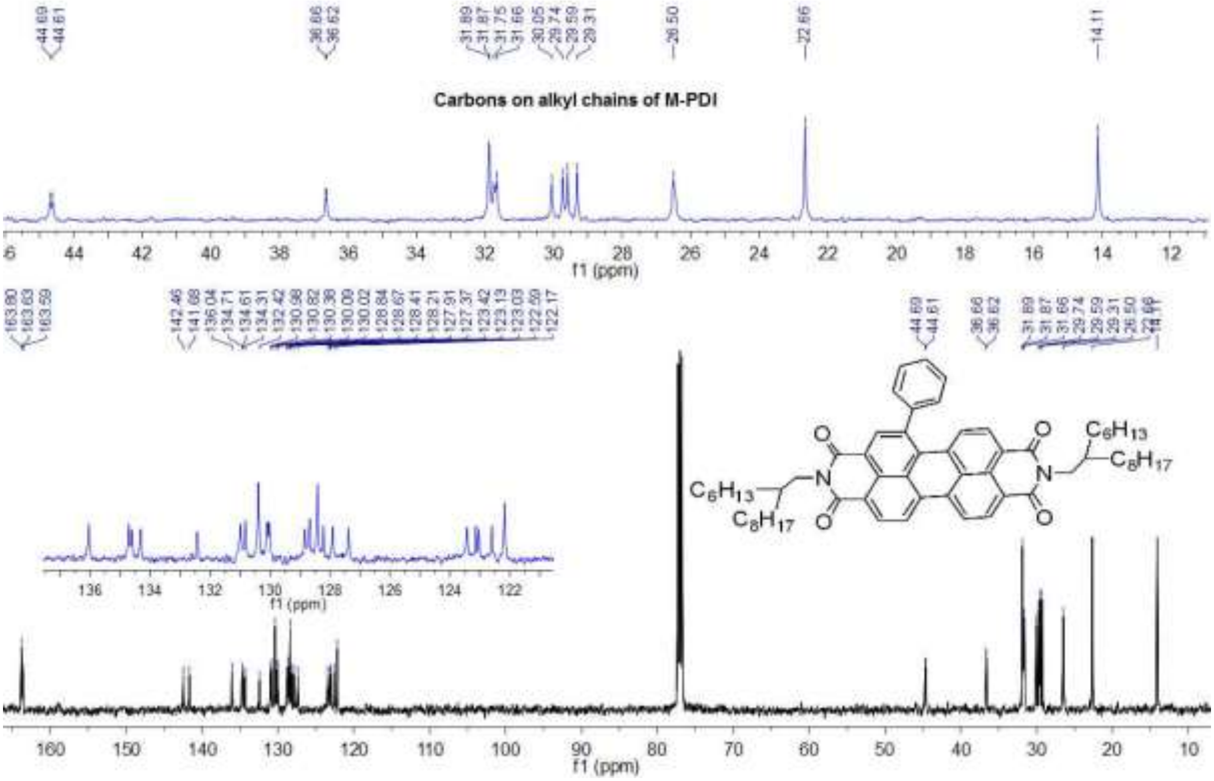

Figure S17. ^{13}C NMR of Mono-PDI.

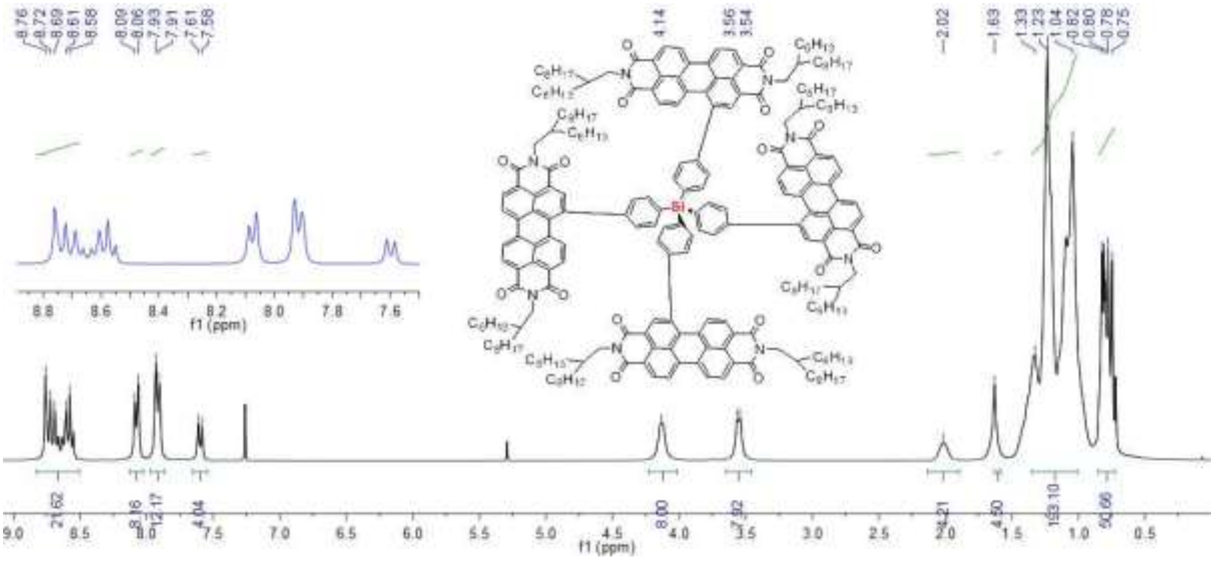

Figure S18.^{1}H NMR of Tetra-PDI.

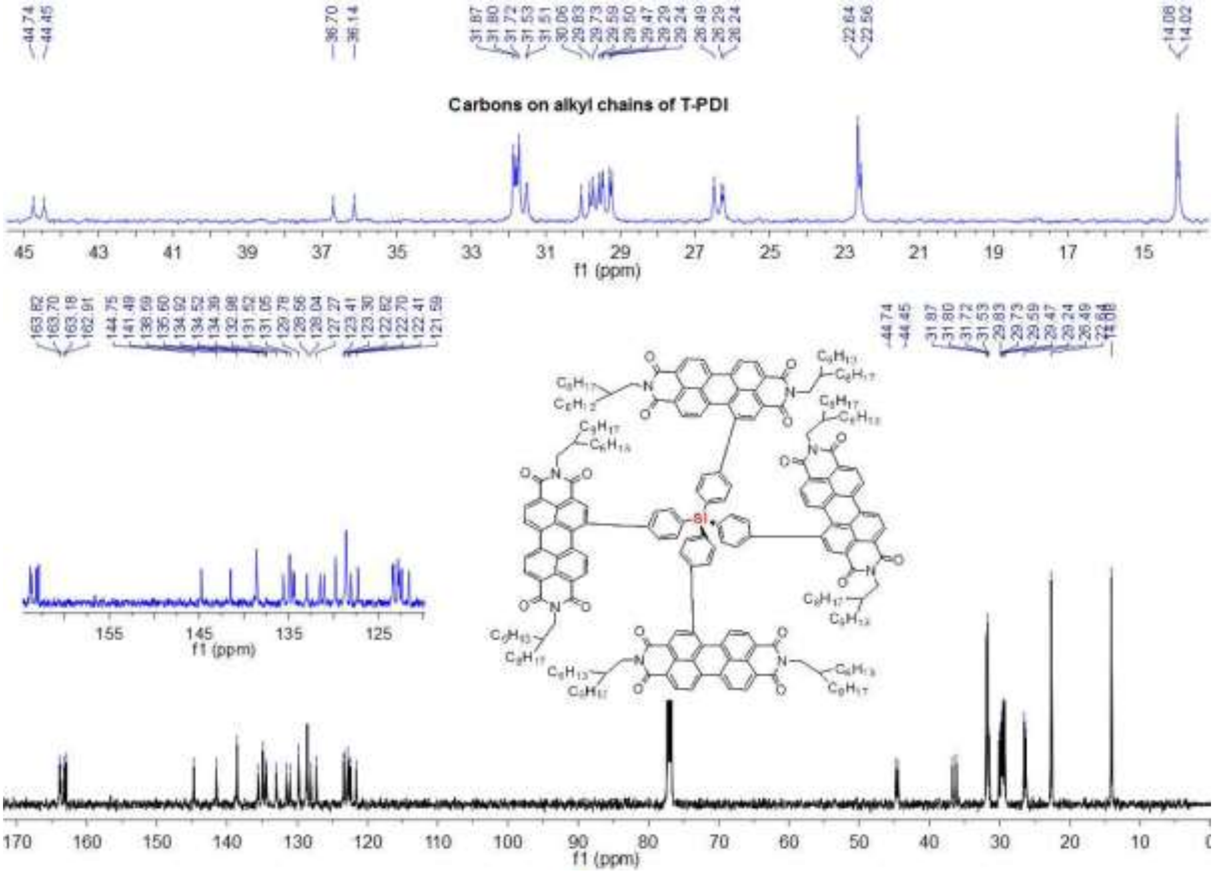

Figure S19.^{13}C NMR of Tetra-PDI.

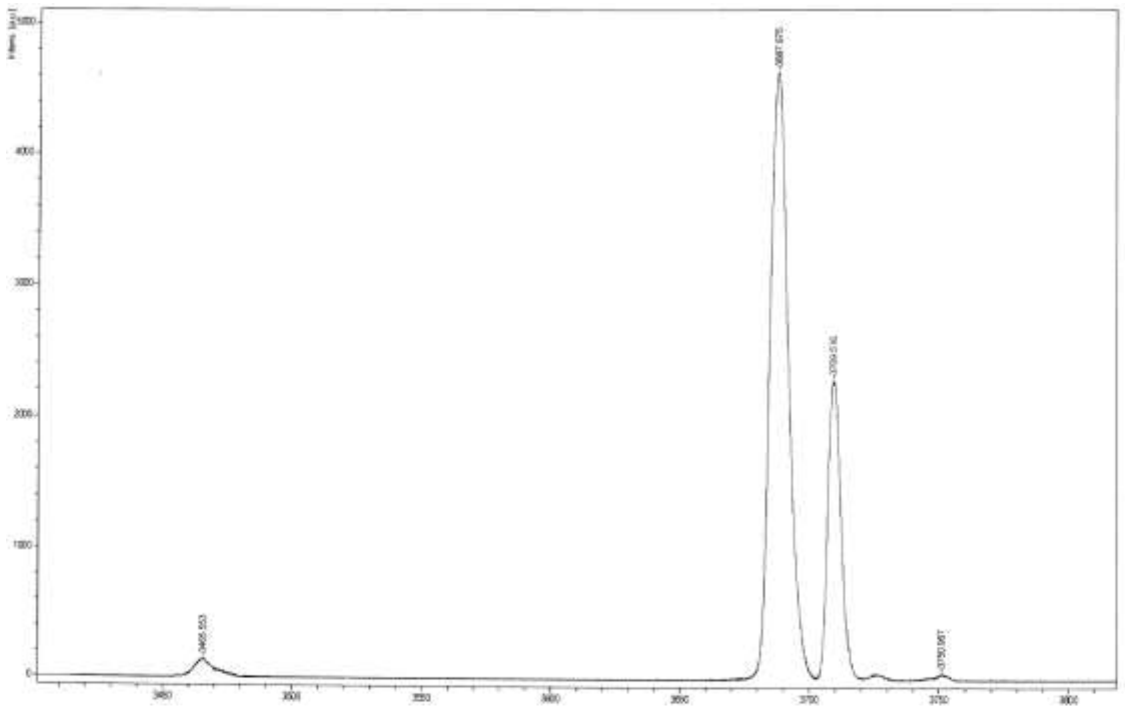

Figure S20. MALDI-TOF MS spectrum of Tetra-PDI.

References

[S1] M. J. Frisch, *et al. Gaussian, Inc., Wallingford CT,* **2009**.

[S2] J.-H. Fournier, X. Wang, J. D. Wuest, *Can. J. Chem.*, **2003**, *81*, 376.

[S3**]** X. Guo, M. D. Watson, *Org. Lett.*, **2008**, *10*, 5333.

[S4] Z. Chen, B. Fimmela, F. Würthner, *Org. Biomol. Chem.*, **2012**, *10*, 5845.

[S5] P. Rajasingh, R. Cohen, E. Shirman, L. J. W. Shimon, B. Rybtchinski, *J. Org. Chem.*, **2007**, *72*, 5973.
